# Development of Antimicrobial Phototreatment Tolerance: Why the Methodology Matters

**DOI:** 10.3390/ijms22042224

**Published:** 2021-02-23

**Authors:** Aleksandra Rapacka-Zdonczyk, Agata Wozniak, Joanna Nakonieczna, Mariusz Grinholc

**Affiliations:** 1 Laboratory of Molecular Diagnostics, Intercollegiate Faculty of Biotechnology, University of Gdansk and Medical University of Gdansk, Abrahama 58, 80-307 Gdansk, Poland; aleksandra.rapacka-zdonczyk@ug.edu.pl (A.R.-Z.); agata.wozniak@phdstud.ug.edu.pl (A.W.); joanna.nakonieczna@biotech.ug.edu.pl (J.N.); 2 Department of Pharmaceutical Microbiology, The Faculty of Pharmacy, Medical University of Gdansk, Hallera 107, 80-416 Gdansk, Poland

**Keywords:** antimicrobial blue light, antimicrobial photodynamic inactivation, cold atmospheric plasma, pulsed light, persistence, resistance, tolerance, ultraviolet light

## Abstract

Due to rapidly growing antimicrobial resistance, there is an urgent need to develop alternative, non-antibiotic strategies. Recently, numerous light-based approaches, demonstrating killing efficacy regardless of microbial drug resistance, have gained wide attention and are considered some of the most promising antimicrobial modalities. These light-based therapies include five treatments for which high bactericidal activity was demonstrated using numerous in vitro and in vivo studies: antimicrobial blue light (aBL), antimicrobial photodynamic inactivation (aPDI), pulsed light (PL), cold atmospheric plasma (CAP), and ultraviolet (UV) light. Based on their multitarget activity leading to deleterious effects to numerous cell structures—i.e., cell envelopes, proteins, lipids, and genetic material—light-based treatments are considered to have a low risk for the development of tolerance and/or resistance. Nevertheless, the most recent studies indicate that repetitive sublethal phototreatment may provoke tolerance development, but there is no standard methodology for the proper evaluation of this phenomenon. The statement concerning the lack of development of resistance to these modalities seem to be justified; however, the most significant motivation for this review paper was to critically discuss existing dogma concerning the lack of tolerance development, indicating that its assessment is more complex and requires better terminology and methodology.

## 1. Introduction

Increasing antimicrobial resistance due to overuse and misuse of antibiotics is a significant concern and extremely dangerous health threat facing modern medicine [1]. Bacteria generally, but species defined with the acronym ESKAPE (*Enterococcus faecium*, *Staphylococcus aureus*, *Klebsiella pneumoniae*, *Acinetobacter baumannii*, *Pseudomonas aeruginosa*, and *Enterobacter* spp.) in particular, have continually evolved a repertoire of evasive mechanisms to defy antibiotics [2]. Nowadays, about 700,000 people die every year due to infections caused by multidrug-resistant pathogens [3]. Thus, there is an urgent need to develop new alternative therapeutic strategies to treat drug-resistant infections for which there is a low risk of developing resistance [4]. Regarding antimicrobial photodynamic inactivation (aPDI) and antimicrobial blue light (aBL) treatments, there is considered to be a low risk of microbial tolerance or resistance developing due to the multitarget mode of action of these treatments. After conducting a critical review, we describe here recently published studies concerning the risk of developing tolerance and resistance to phototreatments, i.e., antimicrobial photodynamic inactivation, antimicrobial blue light, and other alternative light-based approaches, such as UV irradiation and pulsed light. Furthermore, we propose a protocol to examine potential microbial tolerance and resistance development. We also delineate a framework for classifying the bacterial response to multiple photodynamic modalities (resistance, tolerance, and persistence).

Resistance/tolerance or persistence vs. susceptibility: How to distinguish and properly define these three phenomena.

In the case of light-based treatments, a rigorous distinction between the terms “resistance” and “tolerance” is lacking, which could lead to misclassification of observed phenomena. Resistance, when referring to antibiotics, is defined as an acquired and inherited decline in effectiveness of a given antimicrobial that results in the need for higher concentrations of the drug [5]. According to the Scientific Committee on Consumer Safety (SCCS), “the practical meaning of antibiotic resistance is to describe situations where (i) a strain is not killed or inhibited by a concentration attained in vivo, (ii) a strain is not killed or inhibited by a concentration to which the majority of strains of that organism are susceptible, or (iii) bacterial cells that are not killed or inhibited by a concentration acting upon the majority of cells in that culture” [6].

**Figure 1:**
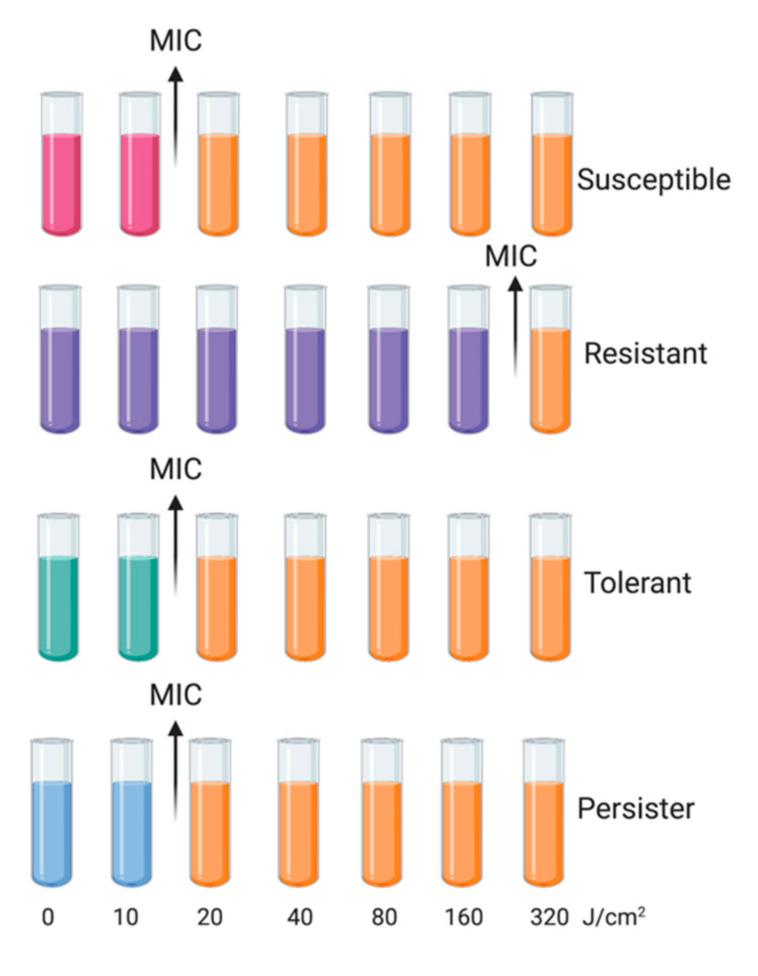
Minimum inhibitory concentration (MIC) characteristics of bacterial responses to light-based treatments. MIC values for bacterial strains resistant to light-based treatment are significantly higher than those for susceptible strains. Resistance is an acquired and inherited decline in the effectiveness of a given treatment (the need for higher concentrations of a photosensitizing agent); Tolerance is an acquired stable feature (the need for longer treatment duration to achieve the same killing efficacy regardless of the concentration of the photosensitizing agent); Persistence is a nonheritable and dormant phenotypic state (transient tolerance) represented by a small subpopulation (about 0.1–1%). Colored probes represent bacterial growth, and orange indicates growth inhibition due to phototreatment conditions leading to cell death. MIC values for strains expressing tolerance or persistence are similar to those of susceptible strains. Concentrations are chosen for illustration purposes only (modified from Brauner et al.).

The level of resistance can be determined using minimum inhibitory concentration (MIC) testing. Tolerance is a more general term that is also connected with the failure of antimicrobial treatment, and it was defined first by Kester and Fortune (2013) and then by Brauner et al. (2016) as the ability of microorganisms (inherited or not) to survive temporary exposure to concentrations of a drug that would otherwise be lethal [5,7]. It can be conferred by environmental conditions (phenotypic tolerance) or through genetic mutations (genotypic tolerance) [8]. In the literature, the terms “resistance” and “tolerance” are often used interchangeably, but they should be distinguished due to different responses to treatment and underlying mechanisms. For clarification, in this review, we define tolerance as inherited reduced efficacy of a treatment. Bacterial strains that develop tolerance can have the same MIC as nontolerant susceptible strains (Figure 1).

For this reason, Fridman et al. (2014) and Brauner et al. (2016) suggested minimal duration for killing 99% of cells (MDK_99_) as a good tool to quantify tolerance. When higher tolerance is observed, a longer treatment duration is needed to reach the same level of killing, which translates to a higher MDK_99_ value [5,9]. The measurement of both parameters, MIC and MDK, could help in evaluating clear differences between resistance (higher MIC) and tolerance (higher MDK) [5].

**Figure 2:**
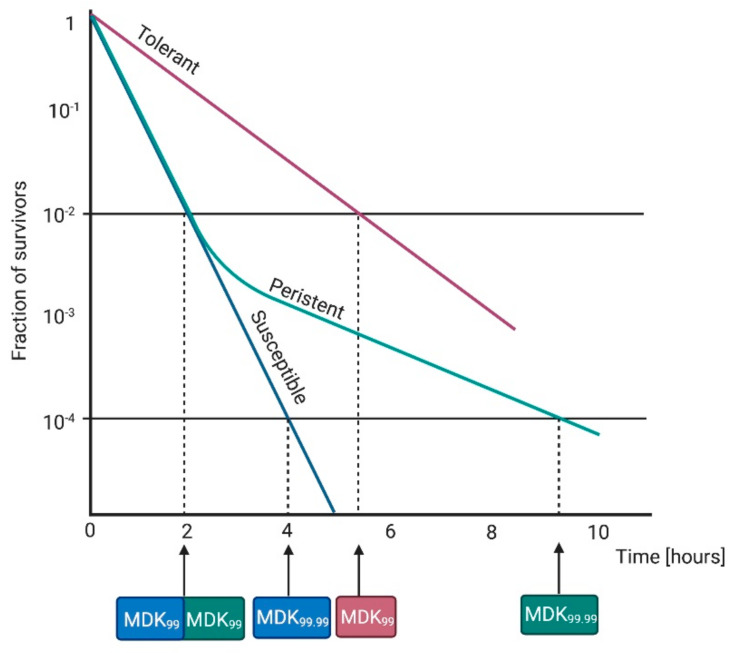
Characteristics of bacterial responses to light-based treatments. Minimum duration for killing 99% of bacterial cells (MDK_99_) is substantially longer for tolerant than susceptible and persistent strains. MDK_99_ for persistent and susceptible strains is similar, but MDK_99.99_ for persistent strains is substantially higher than for susceptible strains. Time scale is chosen for illustration purposes only (modified from Brauner et al.).

Moreover, the whole concept is further complicated by the phenomenon of persistence. The terms “resistance” and “tolerance” can be applied to whole microbial populations, while “persistence” is characteristic of a surviving subpopulation (ranging from 10^−6^ to 10^−1^) and mainly occurs when a majority of bacterial cells is instantly killed [10]. Furthermore, it is nonheritable, and persisters are genetically identical to their nontolerant kin, but with a transient main cellular process blockage [11]. Persisters have a slow or non-replicating growth rate due to forceful environmental stresses, e.g., oxidative stress or starvation [12,13,14,15,16]. Persisters have similar MIC and MDK_99_ values to susceptible cells, but higher MDK_99,99_ (Figure 2) [5].

According to the information described above, with regard to phototreatments, the following terms can be defined:

- Resistance is an acquired and inherited decline in the effectiveness of a given treatment, resulting in the need for high concentrations of a photosensitizing agent and/or longer exposure to the treatment; it should be a stable feature, observed in the next consecutive cycles. In the case of phototreatments, due to the unspecific mechanism, this is rare, and has not been observed to date.
- Tolerance is an acquired stable feature, whereby longer minimum treatment duration (e.g., irradiation time) is needed to achieve the same killing efficacy regardless of the concentration of the photosensitizing agent; it is characterized by being stable and, thus, is observed in subsequent consecutive cycles.
- Persistence is a nonheritable and dormant phenotypic state (transient tolerance) represented by a small subpopulation (about 0.1–1%) of bacterial cells that are killed at a slower rate than susceptible cells [17]; it can be observed as unstable tolerance, occurring in a few cycles of photoinactivation and vanishing in subsequent cycles.

Light-based treatments, due to the nonselective, multitarget, and reactive oxygen species (ROS)-dependent mechanisms of action, are considered unlikely to induce bacterial tolerance and/or resistance. In case of aBL or aPDI, the development of resistance/tolerance has been extensively studied over the past decade [18,19,20,21,22,23,24,25,26,27,28,29,30,31,32,33]. Many of these works were reviewed in depth by Kashef and Hamblin (2017); however, the phenomenon of tolerance/resistance was not observed in any of the publications included in this review [34]. There is no standard protocol to predict the development of bacterial resistance; nonetheless, there are a few possible reasons why cells do not obtain tolerance or resistance to photoinactivation:

(i) Inoculation from a single surviving colony: This procedure is burdened with the risk of very low probability of detecting tolerance or resistance due to the fact that the majority of surviving bacterial cells that could carry genetic alterations are omitted and not included in the next cycle. Single surviving colonies were used as inoculation sources for the next cycle in numerous studies [19,24,27,29,30,32]. However, few studies have described the use of treated suspensions for re-inoculation [20,26,28,31,33,35].
(ii) Application of lethal rather than sublethal doses or irradiation times longer than the minimal duration for killing 99% of cells (MDK_99_): Too-high doses of light-based treatment could lead to irreversible changes in bacterial cells and contribute to the state that most bacterial cells would not be able to recover and form tolerant/resistant phenotypes. Most of the published studies describe the use of lethal instead of sublethal conditions [19,20,21,25,29,32,36]. Sublethal treatment was applied in just a few studies [18,24,26,27,28,30].
(iii) Too few consecutive passages (<15 cycles).
(iv) Lack of verification regarding whether the adaptation is stable and the change has a genetic basis or is due to persistence. In a study by Zhang et al. (2016), reduced aBL susceptibility with increasing number of cycles (fourth and fifth passages) was observed in *Candida albicans*. However, there was no statistically significant difference in the post-aBL survival rate of *C. albicans* between the first and last passage (P > 0.05). Leanse et al. (2018) observed an unstable decrease in aBL efficacy in *A. baumannii* in the 9th, 16th, and 17th cycles. The temporary, unstable reduction of susceptibility observed in these two studies may indicate the appearance of persister cells due to oxidative stress and phenotype switching [12]; thus, it is necessary to validate whether the observed decrease in treatment susceptibility is a stable feature.
(v) Lack of untreated control, in order to exclude the phenomenon of naturally occurring mutations due to cell aging with an increasing number of passages.

A study by Guffey et al. (2013) suggested that *S. aureus* may be capable of developing adaptation to blue light irradiation. Subsequent applications of blue light (405 nm) to subcultured generations of *S. aureus* were increasingly effective through four cycles. Starting from the fifth cycle, a decrease in effectiveness was observed [23].

In turn, in studies performed by Amin et al. (2016), *P. aeruginosa* exhibited reduced susceptibility to sublethal aBL treatment after nine cycles of photoinactivation. The fraction of surviving cells was increased by approximately 2 log_10_ units compared with the first cycle. While the authors did not consider this result as an indication of resistance, we consider that this observation may indicate the possible development of tolerance. 

Our studies indicated the development of *S. aureus* tolerance to RB-mediated aPDI and aBL (405 nm) when reference USA300 JE2 strain was subjected to 15 cycles of sublethal treatment. Potential reductions in susceptibility to aPDI and aBL were examined after the 5th, 10th, and 15th consecutive cycle. Developed adaptation was stable after five cycles of subculturing without aPDI/aBL exposure. The development of aPDI/aBL tolerance was also demonstrated for clinical methicillin-resistant *S. aureus* (MRSA) and methicillin-sensitive *S. aureus* (MSSA) strains as well as other representatives of Gram-positive species, i.e., *Enterococcus faecium* and *Streptococcus agalactiae*. A key point of the results was the lack of cross-tolerance between RB and aPDI mediated by other PSs, i.e., new methylene blue (NMB) and meso-Tetra(N-methyl-4-pyridyl)porphine tetratosylate salt (TMPyP), along with a lack of cross-tolerance between aPDI and aBL. It needs to be highlighted that the developed aPDI/aBL tolerance cannot be considered resistance because more rigorous administration, i.e., increased photosensitizer (PS) concentrations and/or higher light doses, caused bacterial eradication [31].

In our most recent study, we also demonstrated the development of aPDI tolerance. The application of 10 cycles of sublethal aPDI resulted in significant tolerance for all tested *S. agalactiae* strains, including the reference (ATCC 27956) and clinical isolates (strains 2306/06 and 2974/07). The developed tolerance decreased aPDI efficacy up to 3 log_10_ units in viable counts. Moreover, the phenotypic stability of the developed tolerance was observed when samples were passaged for the next five cycles with no selection pressure. The obtained results revealed increased tolerance after passaging, resulting in reduced aPDI efficacy by 5 log_10_ units [33]. The abovementioned studies indicate that the developed adaptation was a result of genetic alterations and may be transferred to subsequent generations without selective pressure. 

The most recent study concerning the tolerance to aPDI was performed by Snell et al. (2021) who revealed that repeated exposure to MB-mediated aPDI lead to increased sur-vival rate of two reference *S. aureus* strains, i.e., HG003 and ATCC 25923, after 7-day-lasting consecutive treatment. These results are contradictory to these published by Pedigo et al. (2009) even though using the same *S. aureus* strains. Moreover, the research by Snell et al. described the application of global transcriptome and genome analysis to identify the essential regulatory and genetic adaptations that contributed to the observed stable tolerance.

**Figure 3:**
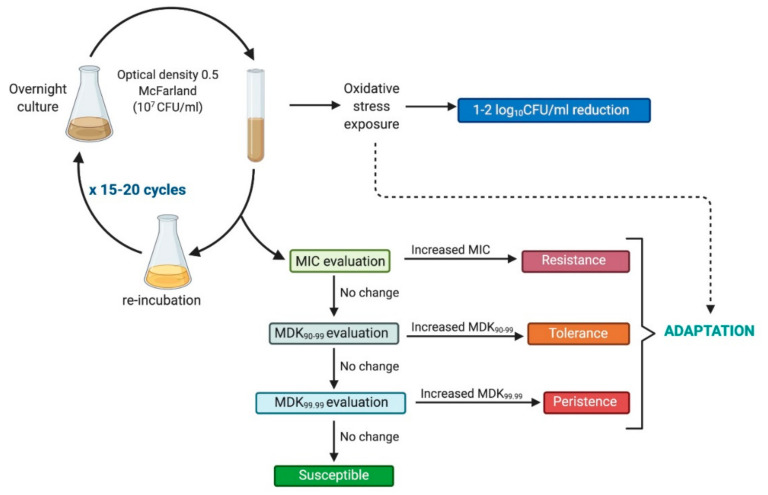
Framework for photoinduced adaptation research.

The observed variations in methodological approaches are the reason why, in our previously published study [31], we proposed the following protocol to determine the risk of developing tolerance/resistance in response to sublethal light-based treatments (Figure 3):

- Exposure of examined strains to phototreatment should result in a 1 to 2 log_10_ reduction in viable counts to leave sufficient survivors for possible development of tolerance, so doses used should be rather close to the MDK_90–99_ parameter [9,37].
- The subculture used in the next cycle should originate from the treated suspension, not from a single surviving colony [38,39].
- The experiment needs to be conducted with up to 15–20 passages of sequential subculturing [37,40].
- Phenotypic stability testing should be performed [37,41].

## 2. State of the Art

A description and discussion of the most recent and noteworthy studies concerning the development of phototreatment tolerance are provided below. More detailed descriptions of the findings are presented in the following tables: Table 1 for aBL and aPDI studies, Table 2 for studies on cold atmospheric plasma, and Table 3 for UV studies. It is worth mentioning that all reviewed light-based strategies meet the basic criteria for being antimicrobial approaches as their employment leads to bacterial viability reduction by more than 3 log_10_ units in viable counts.

### 2.1. Antimicrobial Blue Light (aBL)

Antimicrobial blue light (aBL), is a widely studied bactericidal strategy leading to ac-tivation of endogenously produced photosensitizing molecules, i.e., uro-, coproporphy-rins, flavins etc., via visible light irradiation in the spectrum of 400–470 nm, in particular 405 nm [42]. The hypothesized mechanism of action of aBL involves the microbial en-dogenous accumulation of photoactive compounds that are subsequently excited to the triplet state leading to reactive oxygen species (ROS) generation [43]. Next, ROS react with a wide range of microbial components leading to deleterious effects against various key components such as cellular envelopes, proteins, lipids, and genetic material.

One of the first studies that attempted to demonstrate resistance to aBL by employing naturally and intracellularly occurring photoactive agents, i.e., endogenous porphyrins, was performed and described by Guffey et al. For the experimental procedures, *S. aureus* was used as a reference strain. The bacterial suspension was plated on mannitol salt agar (MSA) medium or blood agar (BA) and then exposed to aBL. Surviving colonies were subcultured on fresh medium, and then a few colonies were used to prepare the inoculum for the next experimental cycle. Overall, the experimental outcome suggested that *S. aureus* developed aBL tolerance, and this phenomenon was observed at the fourth cycle of the experiment. However, the authors did not present any data referring to the control group and nor perform any additional culturing to determine whether the acquired tolerance was stable [23]. The same *S. aureus* strain and methodology were used in another study by Guffey et al. The research objectives included determining whether factors such as light dose, wavelength, etc., would influence the formation of aPDI tolerance. The experiment included seven cycles, and for all of them, the cells that survived aBL/aPDI treatment and grew on solid MSA medium were subcultured on BA and then used in the next experimental stage (for inoculum preparation). At the first stage, cells were irradiated with aBL (450 nm), then with two light wavelengths in the second, fourth, and sixth stages, 464 nm and infrared 850 nm. In the remaining experimental cycles, 464 nm light was used. The various light wavelengths (450, 464, and 850 nm) and fluence rates (125, 20, and 10 mW/cm^2^) were also investigated. The results demonstrated that tolerance developed, suggesting that infrared combined with blue light can delay the occurrence of tolerance [22]. 

Another study concerning aBL tolerance was conducted by Zhang et al., who performed experiments with a multidrug-resistant (MDR) *A. baumannii* clinical isolate. Overnight bacterial culture diluted to a cell density of approximately 10^8^ CFU/mL was placed in a Petri dish and irradiated with aBL. The antimicrobial efficacy of the aBL led to reduced bacterial viability by approximately 4 log_10_ units. Even though the authors defined this as sublethal aBL, based on our knowledge, it should be considered lethal treatment. The experiment involved treating with aBL, plating the surviving colonies on brain heart infusion (BHI) plates, and reculturing them in liquid medium for the next aBL cycle. This procedure was repeated until the 10th consecutive cycle was achieved. The authors reported that after 10 photoinactivation cycles, tolerance did not develop. Moreover, they demonstrated that bacterial cells originating from the first, third, and ninth treatment cycles displayed higher susceptibility to aBL than the parent strain. To explain this phenomenon, the authors supposed that at least one mutation favoring bacterial susceptibility to aBL occurred [24]. The results obtained by Zhang et al. are encouraging; however, these results could have been affected by the application of lethal rather than sublethal aBL. Similarly, Amin et al. investigated the development of aBL by using *P. aeruginosa* as a model organism. Bacteria were subjected to 10 sublethal aBL cycles, and no tolerance to aBL was detected, indicating that the same aBL effectiveness of the parent and *P. aeruginosa* originated from the next cycles of aBL treatment [26].

Another study, conducted by Tomb et al., investigated the effect of repeated exposure of methicillin-sensitive and methicillin-resistant *S. aureus* to sublethal, high-intensity 405 nm light. Overnight cultures of MRSA and MSSA were diluted to an optical density of 10^5^ CFU/mL and exposed to aBL. The applied light dose led to reduced MSSA and MRSA viability by 1.3 log_10_. After exposure, cells were plated on nutrient agar (NA) and surviving colonies were used for inoculation of liquid nutrient broth (NB) for the next experimental cycle. The experimental procedure consisted of 15 exposure–subculture–exposure cycles. Apart from aBL susceptibility testing, the kinetics of the surviving cells’ response to aBL treatment was evaluated for cells originating from the 5th, 10th, and 15th cycles. Repeated sublethal exposure revealed fluctuations in MSSA viable cell reduction; however, there were no significant differences when compared with untreated control, indicating *S. aureus* did not develop tolerance to aBL [27]. 

A study by Leanse at al. (2018) also examined the possible development of aBL tolerance in clinical isolates of three Gram-negative bacterial species: *A. baumannii*, *P. aeruginosa*, and uropathogenic *E. coli*. Bacterial suspensions containing 10^7^ CFU/mL were exposed to aBL, reaching 4 log_10_ unit reduction in viable counts. Single surviving colonies were used to inoculate the suspension for the next cycles. The experiment consisted of 20 consecutive cycles. In addition to in vitro testing, the potential for developing tolerance was also investigated in vivo, using a mouse model of wounds infected with a bioluminescent strain of *P. aeruginosa*. After five consecutive applications of aBL, the bacteria were isolated from the infection site and subjected to aBL treatment in vitro. No statistically significant change in bacterial susceptibility to aBL was observed in either in vivo or in vitro assays. Nevertheless, unexpected variability in log_10_ unit reduction was found to occur within individual cycles of exposure, in which cells displayed more tolerant phenotypes compared with control samples. However, the feature was unstable, and the bacteria reverted to being sensitive when cultured with no selective pressure. We speculate that the lack of developed tolerance could have been affected by the light dose, resulting in lethal rather than sublethal bacterial inactivation [29]. 

Another study concerning potential development of *C. albicans* tolerance to aBL was performed by Zhang et al. (2016). In this study, a suspension containing 10^7^ CFU/mL of *C. albicans* was exposed to aBL (415 nm), resulting in cell viability being reduced by 5 log_10_ units, which indicates the treatment should be considered lethal. Surviving colonies were recultured for the next cycle. The procedure was repeated for 10 consecutive passages. aBL serial exposure revealed a tendency of reduced *C. albicans* susceptibility to aBL starting from the 4th and 5th passages, but no statistically significant differences were found when comparing the survival rate from the 1st to the 10th passage [25]. 

Finally, our research group also recently published studies showing that sublethal aBL treatment leads to the development of tolerance in *S. aureus* and two other Gram-positive bacterial species [31]. The reference *S. aureus* strain, diluted to an optical density of approximately 10^7^ CFU/mL, was irradiated with 411 nm light, leading to cell viability being reduced by 2 log_10_ units. Following exposure, treated suspensions were transferred to fresh tryptic soy broth (TSB) medium to regrow overnight. The next day, the treatment was repeated under the same conditions. The cycle of exposure–regrowth–exposure was repeated 15 times. Potential reduction in the susceptibility to aBL was examined after the 5th, 10th, and 15th consecutive cycles. A 2 log_10_ unit decrease in aBL antimicrobial efficacy was observed starting from the 5th cycle. Intriguingly, the developed aBL tolerance was found to be associated with reduced *S. aureus* susceptibility to H_2_O_2_ and an increased mutation rate, and also had an impact on the antimicrobial susceptibility of *S. aureus* to specific antimicrobials.

### 2.2. Antimicrobial Photodynamic Inactivation (aPDI)

Antimicrobial photodynamic inactivation (aPDI) is a new strategy to killing infectious pathogens. aPDI is based on excitation of a dye molecule (called a photosensitizer, PS) by visible light. The excited PS forms a triplet state that can react with oxygen to pro-duce ROS. These ROS may exert its activity towards essential biomolecules, i.e., lipids, proteins, nucleic acids, leading to microbial cell lysis and death. There is a wide variety of PS structures that have been demonstrated to be effective against wide range of pathogens, including xanthenes such as rose Bengal (RB), phenothiazinium salts like methylene blue (MB), and tetrapyrrole structures, i.e., porphyrins, and phthalocyanines.

#### 2.2.1. Xanthene Photosensitizers

According to our recently published studies concerning the development of aBL tolerance, we also observed the development of significant tolerance in *S. aureus* exposed to repetitive Rose Bengal (RB)–aPDI treatment for 15 cycles. The use of RB photoactivated with green light resulted in the development of tolerance, with lower susceptibility to RB–aPDI starting from the fifth consecutive cycle, expressed as a reduction in aPDI antimicrobial efficacy by approximately 3 log_10_ units (notably, the significant reduction in *S. aureus* susceptibility to RB–aPDI was observed from the third consecutive cycle). In both cases, aPDI and aBL, the developed tolerant phenotype was stable under cultivation with no selective pressure [31].

Pieranski et al. (2020) demonstrated the development of aPDI tolerance in group B *Streptococcus* (GBS). For this study, sublethal conditions of RB-mediated aPDI treatment leading to an approximately 1 log_10_ unit reduction were selected. Irradiation with 515 nm light in the presence of RB for 10 consecutive cycles resulted in the development of significant tolerance for the tested reference and clinical *S. agalactiae* isolates. The developed tolerance manifested as a decline in aPDI effectiveness up to 3 log_10_ units in CFU/mL. The phenotypic stability of the developed tolerance was also tested. *S. agalactiae* cultures originating from the 10th consecutive cycle that expressed notable aPDI tolerance were passaged for the next five cycles without selective pressure. Then, the susceptibility of *S. agalactiae* cultures subjected to aPDI was evaluated over these passages. No loss of developed aPDI tolerance was observed. The results indicated increased tolerance after passaging with aPDI efficacy reduced by 5 log_10_ units. These results support the assumption that the acquired adaptation resulted from genetic alterations and could be transferred to subsequent generations without selective pressure. 

Sequencing of 1525 bp upstream DNA fragments of one of the *cylE* genes (responsible for oxidative stress response within *S. agalactiae*) revealed that the functionality of the *cyl* operon changed distinctly over the process of sublethal aPDI treatment. The results suggested that 10 cycles of aPDI treatment led to an increase in single mutation events. Next, the aPDI-tolerant *S. agalactiae* (after 10 consecutive treatments with sublethal aPDI) was exposed to five oxidants: hydrogen peroxide (H_2_O_2_), paraquat (superoxide), hypochlorite, new methylene blue (NMB) (singlet oxygen), and zinc phthalocyanine (ZnPc) (a structurally different PS from RB). aPDI-tolerant *S. agalactiae* showed no change in susceptibility to these oxidants (excluding hydrogen peroxide; increased tolerance of aPDI-tolerant cells to H_2_O_2_ was observed). Furthermore, the results indicate no cross-tolerance. Similar to the results obtained by Rapacka-Zdonczyk et al. (2019), RB–aPDI treated cultures exhibited even higher susceptibility to NMB-mediated aPDI. The results also revealed that upon sublethal treatment, there were direct changes in the expression levels of stress-related genes. Increased expression of *sodA*, *tpx*, and *recA* and decreased expression of *ahpC* and *cylE* were reported. Notably, for strains treated with 10 cycles of sublethal aPDI, expression without aPDI treatment was significantly increased for all tested stress-related genes. Afterward, the aPDI-tolerant strain treated with sublethal aPDI had significantly increased expression of *sodA, npx, tpx,* and *recA*. Moreover, morphological changes in *S. agalactiae* colonies occurred. Compared with the wild-type strain, the aPDI-tolerant strain exhibited different proportions of unpigmented and dark colonies. Similarly, the aPDI-tolerant strain exhibited increased hemolytic properties. As expected, the use of an adequate experimental protocol described by Rapacka-Zdonczyk et al. (2019) demonstrated that multiple sublethal aPDI treatments led to the development of considerable and phenotypically stable tolerance in *S. agalactiae*.

#### 2.2.2. Phenothiazine Photosensitizers

For phenothiazines, the application of sublethal methylene blue (MB)-aPDI to *E. coli* (ATCC 25922) cells for 11 consecutive cycles and to MRSA (ATCC 33592) and MSSA *S. aureus* (ATCC 25923) strains for 25 cycles was investigated. After aPDI application, microorganisms were incubated for 48 h, followed by cell viability testing. The experimental outcome indicated that repetitive sublethal MB–aPDI treatment did not lead to significant changes in bacterial susceptibility to phototreatment. However, we note that the 48-h time period used for incubation could have significantly affected the overall result, being sufficiently long for recovered cells to effectively eliminate tolerant cells from the population [18]. In addition, the authors did not define the sublethal dose, but the applied doses seemed to be far more bactericidal, achieving a 2 log_10_ unit reduction in viability; thus, in our opinion, this could also have affected the results as the light dose could be defined as lethal. 

The opposite results were described very recently by Snell et al. (2021) who demonstrated the development of aPDI tolerance in case of two *S. aureus* strains, i.e., HG003 and ATCC 25923 (comparative control), which were previously used by Pedigo et al. and re-ported as not developing tolerance to MB-mediated aPDI (18). *S. aureus* strains (7 × 10^8^ CFU/mL) were subjected to sublethal aPDI. Treated suspensions were cultured overnight and proceeded for a next subsequent aPDI cycle. After completion of seven consecutive aPDI cycles, both *S. aureus* strains exhibited stable tolerance. Moreover, the performed study demonstrated the occurrence of cross-tolerance to other, structurally similar, phe-nothiazine PS, i.e., toluidine blue O (TBO)-mediated aPDI. Additionally, study by Snell et al. described the application of transcriptional and genomic analysis and demonstrated that the multiple metabolic pathways, cell wall biogenesis, DNA recombination and re-pair are associated with observed aPDI tolerance [35].

#### 2.2.3. Phthalocyanines 

A study by Giuliani et al. described the use of single bacterial or fungal colonies to prepare the inoculum for multistep resistance selection. The bacterial and fungal colonies were selected from those that survived the aPDI procedure using tetracationic Zn(II) phthalocyanine chloride (RLP068/Cl) at a concentration corresponding to the minimal bactericidal concentration (MBC). Overnight cultures of *S. aureus* and *P. aeruginosa*, resuspended to obtain a final inoculum density 10^8^ CFU/mL, were administered with RLP068/Cl and, after 5 min of incubation, were exposed to light irradiation. After illumination was completed, a 10 µL aliquot was taken for further cycles and serial dilution was then performed to determine the reduction in bacterial viability. Every five cycles, the MCB for aPDI treatment with RLP068/Cl was examined for all tested bacterial strains. The results demonstrated that in the course of 20 consecutive cycles of aPDI treatment, the MCB values were not affected, indicating no development of tolerance. Indeed, after repetitive aPDI exposure, both bacterial species displayed significantly higher tolerance to photosensitizer in dark control than parent strains; however, when exposed to light activation, tolerance could not be observed. These tolerant cells remained stable after 10 days of passaging without selective pressure, indicating that there could have been acquired tolerance; nevertheless, the overall experimental outcome led to the conclusion that aPDI does not generate resistance and/or tolerance [20].

The first report describing fungal tolerance was a study by Giuliani et al. (2010) using two *C. albicans* strains. Fungal colonies used for inoculum preparation were selected from those that survived the aPDI using tetracationic Zn(II) phthalocyanine chloride (RLP068/Cl) at a concentration corresponding to the minimal fungicide concentration (MFC). The authors indicated that the aPDI conditions resulted in viability being reduced by more than 3 log_10_ units, which corresponds to lethal treatment. After 24 h of incubation, cells were centrifuged, washed with phosphate-buffered saline (PBS), and resuspended in PBS to obtain a final inoculum of approximately 10^6^ CFU/mL. Next, cells were administered RLP068/Cl and exposed to light irradiation. Every five cycle, the MCF for aPDI treatment with RLP068/Cl was examined, and the results demonstrated no development of tolerance in either yeast strain (20). 

#### 2.2.4. Porphyrins and Porphyrin Derivatives

In another study by Lauro et al., two anaerobes, *Peptostreptococcus micros* and *Actinobacillus actinomycetemcomitans*, were exposed to porphycene–polylysine conjugates and light. The aPDI protocol included two photosensitizers, 2,7,12,17-tetrakis(2-methoxy-ethyl)-9-glutaramidoporphycene (GlamTMPn) and 2,7,12,17-tetrakis(2-methoxyethyl)-9- p-carboxybenzyloxyporphycene. aPDI treatment of *P. micros* and *A. actinomycetemcomitans* using both PSs led to a reduction in viability by 5–7 log_10_ units, indicating clear lethal exposure. For the tolerance study, both species and both PSs were used. After PS administration and light irradiation, samples were serially diluted and plated on solid agar medium. After anaerobic culturing, the colonies were collected from the plates, adjusted to appropriate optical density, and used for the next photoinactivation cycle. The experiments indicated no development of tolerance; however, we note that the use of rigorous lethal treatment could have affected the obtained results [36].

Another study by Tavares et al. used planktonic cultures of *E. coli* and *Vibrio fischeri* in combination with PS, i.e., Tri-Py+-Me-PF (5,10,15-tris(1-methylpyridinium-4-yl)-20-(pentafluorophenyl)-porphyrin triiodide), activated with white light. After aPDI exposure leading to high bactericidal efficacy (approximately 1 log_10_ of surviving bacteria), the surviving bacterial cells were plated on solid medium and incubated in specific regimens according to species requirements. After incubation, single colonies were selected, inoculated in liquid medium, and administered Tri-Py+-Me-PF. Next, cells were exposed to light, and the overall experimental procedure was repeated 10 times. There was no indication that tolerance developed; however, the use of two different experimental approaches could have affected the obtained results, i.e., the use of lethal rather than sublethal treatment, and the selection of single colonies for the inoculum preparation rather than the suspension aliquot [19].

Next, Paronyan et al. performed a study of the possible development of tolerance in *S. aureus* and *E. coli* using porphyrin derivative tetracationic Zn-mesotetra-[4-N-(2′-butyl) pyridyl]porphyrin (Zn-TBut4PyP) and white light irradiation. The inoculation for repetitive aPDI treatment was performed using single bacterial colonies selected from the solid medium that survived aPDI treatment, leading to a reduction in viability by 3 log_10_ units. For each strain, 10 consecutive aPDI cycles were performed, and the possible development of tolerance was investigated by measuring the MBC of bacterial suspensions originating from each single cycle. Similarly, with the use of lethal treatment and using single-colony inoculation, no development of tolerance as expressed by significant MBC change was observed [32]. 

It is well known that photoinactivation is an effective strategy against microorganisms and tumors. However, numerous published studies have also demonstrated its killing efficacy toward viruses, yeasts, and parasites. Unfortunately, there are limited studies concerning the possible development of tolerance within these microorganisms; nevertheless, some researchers have made an effort to evaluate this phenomenon. A study by Costa et al. evaluated the possible development of tolerance using T4-phage like bacteriophages treated with tricationic porphyrin 5,10,15-tris(1-methylpyridinium-4-yl)-20-(pentafluorophe-nyl)porphyrin tri-iodide (Tri-Py+-Me-PF) and white light. The tolerance study protocol consisted of 10 consecutive cycles, and the results indicated that no change in phage susceptibility was observed [21].

#### 2.2.5. Other Photosensitizing Compounds

A study by Freitas et al. described research on the potential development of tolerance in *E. faecalis* upon Ce6 chlorin- and MB-mediated aPDI administration. The light doses and PS concentrations were selected to reduce survival rates by 1–3 log_10_ units, meeting the requirements of sublethal treatment; however, we noted that single surviving colonies were selected for the next consecutive cycle, which could explain why no tolerance was developed [30]. 

**Table 1:** Antimicrobial blue light (aBL) and antimicrobial photodynamic inactivation (aPDI) studies.

Ref.	Species	PS	Light Source	Methodology	Log_10_ Reduction	Inoculation Source	Tolerance
Bacteria							
[36]	*P. micros* *A. actinomycetemcomitans*	GlamTMPn BOTHMPn	4 × 250 W tungsten lamps	10 cycles	Lethal ^a^	Solid agar plates	No
[18]	*E. coli* *S. aureus*	MB	Non-thermal diode laser, 670 nm	11 cycles *(E. coli)* 25 cycles *(S. aureus)*	Sublethal	Surviving colonies from previous cycle	No
[19]	*V. fischeri* *E. coli*	Tri-Py+-Me-PF	13 × 18W OSRAM 21 lamps, 380–700 nm	10 cycles	Lethal	Single colony survivors from previous treatment used further as overnight culture	No
[20]	*S. aureus* *P. aeruginosa*	RLP068/Cl	Non-coherent halogen lamp, 600–700 nm	20 cycles	Lethal	From each sample, 10 µL was subcultured to perform subsequent cycles	No
[23]	*S. aureus*	-	SLD light probe/405 nm	7 cycles	No data	Irradiated cells were growing in solid medium, then subcultured on another fresh medium and finally used for next experimental stage	Yes
[22]	*S. aureus*	-	Tri-wave light ultrasound device, 464 nm, 850 nm; SLD light probe, 405 nm	7 cycles	No data	Irradiated cells grown in solid medium then subcultured on another fresh medium and used for next experimental stage	Yes
[24]	*A. baumannii*	-	Omnilux clear-U light- emitting diode array, 415 nm	10 cycles	Sublethal	Surviving bacterial cells from agar were collected and recultured for next cycle	No
[26]	*P. aeruginosa*	-	LED, 415 nm	Irradiation of Petri dish containing bacterial suspension (10 cycles)	Sublethal and lethal	Treated suspension	No (after 9th cycle surviving fraction was increased by 2 log_10_)
[27]	*S. aureus*	-	LED, 405 nm	15 cycles	Sublethal	Surviving colonies	No
[28]	*S. aureus* *E. coli*	ZnTnHex-2-PyP	Overhead projector OHP-3100 p, broad spectrum	Continuous growth under sublethal conditions for 48 h (10–20 cycles)	Sublethal	Treated suspension	No
[29]	*P. aeruginosa* *A. baumannii* *E. coli*	-	LED, 405 nm	20 cycles	Lethal	Single surviving colony	No (increased unstable aBL tolerance in 9th, 16th, and 17th cycles)
	*P. aeruginosa* (mouse skin abrasion wounds)	-	-	5 cycles	Sublethal	-	No
[30]	*E. faecalis*	Ce6 MB	No data	4 cycles	Sublethal and lethal	Single surviving colony from agar plate	No
[31]	*S. aureus*	RB -	LED, 515 nm LED, 411 nm	15 cycles	Sublethal	Treated suspension	Stable tolerance to RB-aPDI and aBL
[33]	*S. agalactiae*	RB	LED, 515 nm	15 cycles	Sublethal	Treated suspension	Stable tolerance
[32]	*S. aureus* *E. coli*	Zn-TBut4PyP	Tungsten lamp, 320–780 nm	10 cycles	Lethal	Single surviving colony	No
[35]	*S. aureus*	MB	Broadband visible light, 575–700 nm	7 cycles	Sublethal	Treated suspension	Stable tolerance (cross-tolerance to TBO-aPDI)
**Viruses**							
[21]	T4-like phage	Tri-Py+-Me-PF	13 OSRAM 21 fluorescent lamps (18 W), 380–700 nm	10 cycles	Lethal	Phage suspension prepared from previous experimental cycle sample	No
**Yeast**							
[25]	*C. albicans*	-	LED, 415 nm	10 cycles	Lethal	Surviving cells	Reduced aBL susceptibility with increasing number of cycles
[20]	*C. albicans*	RLP068/Cl	Non-coherent halogen lamp, 600–700 nm	20 cycles	Lethal	From each sample, 10 µL was subcultured to perform subsequent cycles	No

^a^ Cell viability reduction by 1–2 log_10_ (sublethal treatment) and by >3 log_10_ (lethal treatment). PS, photosensitizer; Ce6, chlorin chlorin-e6; GlamTMPn, (2,7,12,17-tetrakis(2-methoxy- ethyl)-9-glutaramidoporphycene); MB, methylene blue; RB, Rose Bengal; RLP068/Cl tetracationic Zn(II) phthalocyanine chloride; TMP, meso-tetra (N-methyl4-pyridyl) porphine tetra tosylate; Tri-Py+-Me-PF, tricationic porphyrin 5,10,15-tris(1-methylpyridinium-4-yl)-20-(pentafluorophe-nyl)porphyrin tri-iodide; Zn-TBut4PyP, tetracationic Zn-mesotetra-[4-N-(2‘-butyl) pyridyl]porphyrin; ZnTnHex-2-PyP, Zn(II) mesotetrakis (N-n-hexylpyridinium-2-yl)porphyrin.

### 2.3. Pulsed Light

Pulsed light (PL) emitted from xenon lamps is characterized by high power and usually consists of wavelengths ranging from UVA to near infrared (NIR), but is also rich in short UVC wavelengths [44,45]. The mechanism of PL activity is still unknown, but various mechanisms have been proposed to explain its lethal efficacy. All of these are related to irradiation from the UV part of the spectrum, along with the photochemical and/or photothermal effect [46]. Despite the lack of knowledge, Bhaya et al. attempted to investigate whether PL could serve as a tool for eradication of food-borne pathogens such as *Listeria monocytogenes*, *E. coli*, *Salmonella* spp., and *Campylobacter jejuni* [45].

Research by Heinrich et al. aimed at investigating whether *L. monocytogenes* would develop tolerance during long-term repeated PL exposure. Overnight cultures were diluted and spread onto solid medium plates at a density of approximately 10^6^ CFU/cm^2^, and exposed to PL irradiation. After 48 h of incubation, the grown colonies were counted and CFU survival rate was estimated. One surviving colony was then selected for the next experimental cycle. This experimental procedure was repeated for 20 cycles for two *L. monocytogenes* strains. The results demonstrated that both *L. monocytogenes* strains exposed to repetitive PL treatment developed tolerance and that this phenomenon seemed to be time dependent. Another study by Massier et al. described the application of sublethal PL to investigate whether *P. aeruginosa* could adapt to further lethal PL treatment. For this purpose, *P. aeruginosa* in the mid-exponential growth phase was exposed to various PL doses. PL conditions that reduced the cell viability by approximately 1 log_10_ were considered sublethal, whereas lethal PL doses resulted in reduced viability by 4–7 log_10_ units. The results showed that sublethal PL may provoke adaptation and tolerance of *P. aeruginosa* to PL. The study was not aimed at testing stability, but it indicated how fast bacterial cells can adapt to PL. The authors suggested that the adaptation was connected to the production of proteins involved in chaperone mechanisms and was probably a response to DNA damage [47].

Another study showing the effectiveness of PL was described by Gomez-Lopez et al., who performed research using various microorganisms (*Candida lambica*, *Bacillus cereus*, and *Clostridium perfringens*), though the possibility of tolerance development was only tested for *L. monocytogenes*. Overnight cultures were diluted, spread onto NA plates, exposed to PL, and then covered with aluminum foil and incubated for 48 h. After incubation, the surviving colonies were selected for the next PL cycle. The experimental procedure consisted of 13 consecutive cycles using PL conditions that resulted in cell viability being reduced by 3 log_10_ units. The results revealed that no PL tolerance was developed [46].

A study by Uesugi et al. investigated the activity of repetitive PL exposure against *L. monocytogenes*, *L. innocua*, and *E. coli*. Overnight bacterial cultures were diluted to an optical density of approx. 10^8^ CFU/mL and exposed to PL. Next, surviving colonies were counted, recultured onto fresh solid medium, and after 24 h of incubation, used to prepare the inoculum for the next PL treatment. The experimental procedure consisted of 10 consecutive cycles. The results demonstrated that none of the tested species developed PL tolerance. We noted, though, that the applied methodology might not have captured sublethally injured cells that might be able to recover during the isolation and regrowth with no selective pressure [48]. A study by Rajkovic et al. used multiple-strain cocktails of *L. monocytogenes*, *C. jejuni*, and *E. coli* for PL exposure. Overnight cultures of four *L. monocytogenes*, three *E. coli*, and four *C. jejuni* strains were mixed to reach cell density of approx. 10^8^ CFU/mL for *C. jejuni* and 10^9^ CFU/mL for *L. monocytogenes* and *E. coli*. Next, suspensions were diluted to a final density of 10^7^ CFU/mL and subjected to sublethal PL, leading to viability being reduced by 1–3 log_10_ units. Surviving cells were subjected to the next PL exposure. The procedure consisted of 20 consecutive cycles. The results demonstrated that *L. monocytogenes* and *E. coli* became more tolerant to PL, reducing its efficacy by 2 log_10_. Moreover, to investigate whether the observed phenotype was a stable feature, the tolerant and susceptible strains were stored for 12 months at −75 °C and then exposed to PL. Tolerance could still be detected, indicating that the developed feature was a stable phenotype [49]. In our opinion it would be beneficial to perform other stability test, i.e., passaging without selective pressure, to confirm that the obtained tolerance is a stable feature.

### 2.4. Cold Atmospheric Plasma (CAP)

Cold atmospheric plasma (CAP) is a partly ionized gas containing free radicals, ions, electrons and photons which are produced due to the electric field action [50,51]. Due to the light component (photons) it may be considered light-based therapy. Moreover, this approach, similarly to other phototreatments, also leads to the disruption and oxygena-tion of multiple cell components, i.e., membranes, lipids etc. [52,53]. The principal mecha-nisms of CAP involve cell membrane permeabilization, activity of reactive oxygen and ni-trogen species as well as the chemical reactions leading to DNA damage [54]. CAP was demonstrated to be an efficient bactericidal treatment leading to Bacillus subtilis and Clos-tridium difficile spores inactivation [55], eradication of MRSA and E. coli in in vitro porcine skin model [56], as well as inactivation of other Gram-positive and Gram-negative species [51,53,57]. A study aimed at investigating the development of CAP tolerance was described by Matthes et al., employing *S. aureus* biofilm culture in medium to mimic an artificial wound environment. Exposure to CAP was performed in a single day with six repeated applications every hour. After 6 h of treatment, treated and control biofilms were dispersed in an ultrasonic bath, and the antimicrobial effect was determined by CFU counting. Each CAP exposure resulted in cell viability being reduced by approximately 1.7 log_10_ units, and no indication of tolerance was observed. Nevertheless, we noted that the cells were not given an opportunity to recover after treatment as 1 h incubation is far too short a period to develop adaptation. The effectiveness of the applied CAP dose seems to be in line with our proposed methodology; however, the fact that biofilms were not able to recultivate for a sufficiently long period could have influenced the final experimental outcome [58]. Another CAP experiment was performed by Zimmermann et al., who used *E. coli* and *Enterococcus mundtii* to study the development of tolerance. Bacterial cultures (10^8^ CFU/mL) were spread on agar plates and exposed to CAP. Overall, four repetitions of the experimental cycle were performed for both strains. The applied CAP dose resulted in a high bactericidal effect, leading to a survivor count of only 10 CFU (viability reduced by approximately 6 log_10_ units). The methodology led to the conclusion that neither species developed CAP tolerance [54]. The most current study by Brun et al. investigated CAP tolerance for *P. aeruginosa* and *S. aureus*. Both strains were exposed to CAP at a cell density of approximately 10^6^ CFU, diluted in Mueller–Hinton broth (MHB) and incubated at 37 °C. Irradiated aliquots were used for the next CAP treatment. All procedures were repeated for seven consecutive cycles. The results did not confirm the development of CAP tolerance, but the lack of a detailed experimental methodology does not enable reliable conclusions to be drawn [50]. 

**Table 2:** Cold atmospheric plasma studies.

Ref.	Species	Plasma Source	Methodology	Log_10_ Reduction	Inoculation Source	Tolerance
[54]	*E. coli* *E. mundtii*	Plasma device (HandPlaSter)	4 cycles	Lethal	Surviving cells on solid medium	No
[58]	*S. aureus*	Radio frequency plasma pen (kinpen09^®^)	6 repetitions for period of 6 h	No data	Same medium with microorganisms for all experimental procedures	No
[50]	*S. aureus* (MRSA) *P. aeruginosa*	Radiofrequency source with helium as working gas	6 cycles	Sublethal *(S. aureus)* Lethal *(P. aeruginosa)*	Same bacterial suspension for all cycles	No

### 2.5. Ultraviolet (UV) Light

The increasing interest in UV light, observed within recent years, concerns particularly food preservation and decontamination. UV light is characterized by strong decontamination properties and high antimicrobial activity. The UV light is categorized into three groups based on different wavelengths: UVA (320–400 nm), UVB (280–320 nm), and UVC (200–280 nm). The latter being the most germicidal due to its direct interaction with genetic material of microbial cells. Indeed, microbial DNA is the primary target for UV light [59]. The main alterations observed in microorganisms induced by UVC and UVB light include thymine dimers and cyclobutane-pyrimidine dimers formation. UVA has been considered to have the least impact on DNA damage due to its indirect photoreactions via singlet oxygen generation [60]. The important changes in DNA after UVA action include production of 8-hydroxy-2′-deoxyguanosine (8-OHdG). All these photoproducts disturb replication and transcription and are responsible for cytotoxic and mutagenic effects in microbial cells [61]. It should be noted that comparing different experimental approaches based on UV light antimicrobial action is made difficult by the fact that different light sources are used. Conventional UV lamps (e.g., mercury vapor lamps) generate light with a wider range, while more modern light sources based on LED (light emitting diodes) systems generate a strictly defined wavelength. The data comparing the use of various light sources is only recently being started to published [62,63].

It is well known that UV light may exert deleterious effects on genetic material, as it is widely absorbed by nucleic acids and, thus, various mechanisms of protection and repair have been developed within microorganisms through the evolutionary process. One of the first reports demonstrating the possible development of UV tolerance was published by Alcantara-Diaz et al., who used *E. coli* for their study. Overnight bacterial cultures were exposed to 80 cycles of growth and irradiation. Cells in the early stationary phase (10^8^–10^9^ CFU/mL) were exposed to UV light from a germicidal lamp (λ 254 nm), immediately diluted in phosphate buffer, and incubated on agar plates with lysogeny broth (LB) medium for 18–24 h. In addition, 0.1 mL aliquot of irradiated sample was transferred to fresh LB medium and incubated for 6 h to reach the early stationary phase and used for the next experimental cycle. The experiment was performed with a starting UV dose of 10 J/m^2^, and after each 10th cycle, the light dose was increased twofold. After completion of 50 and 80 cycles, all bacterial samples demonstrated high tolerance to UV light which, as stated by the authors, probably resulted from mutations in genes responsible for DNA repair and replication [64]. A study by Goldman and Travisano also investigated the possible development of UV tolerance in *E. coli*. Twenty-four single colonies were used to inoculate 24 LB liquid cultures, and 12 were exposed to UV treatment on agar plates. The UV light resulted in cell viability being reduced by approximately 0.6 log_10_ units. The experiments were performed for 60 days, and every 10th day, the stock was prepared from a single surviving colony. In addition, stability and tolerance testing was performed 20 days after the experiment was completed. The obtained data demonstrated significant tolerance development [65]. 

A unique study aimed at UV tolerance involved viruses, and was described by Tom et al. In this study, *E. coli* was a host used for phage growth. The applied UV dose resulted in viral reduction by 4 log_10_. The improved survival of phages compared with parent phages after 30 cycles was reported. The results indicated that phages exposed to 30 cycles of UV exhibited 45-fold improvement in survival rate compared with untreated controls [66]. 

**Table 3:** Ultraviolet treatment.

Ref.	Species	Light Source	Methodology	Log_10_ Reduction	Inoculation Source	Tolerance
Bacteria						
[64]	*E. coli*	15 W Hg vapor UV germicidal lamp, 254 nm	80 cycles	Initially sublethal (increasing 2-fold every 10 cycles)	Cells after irradiation inoculated in fresh LB medium	Yes (resistance)
[46]	*L. monocytogenes*	Xenon flash lamp, spectrum from UV-C to IR	13 cycles	No data/lethal	Single colony survivors on solid medium plate used for preparation of inoculum for next experimental cycle	No
[49]	*E. coli* *C. jejuni* *L. monocytogenes*	Xenon flash lamp, UVC-UV-IR	20 cycles	Sublethal	Cocktail of strains after PL treatment incubated in fresh medium	Yes (resistance)
[65]	*E. coli*	UVP Chromato-Vue TM-36 transilluminator (UVP Inc., Upland, CA, USA), 302 nm	60 cycles	Sublethal	LB agar plates with surviving colonies	Yes (resistance)
[47]	*P. aeruginosa*	4 × xenon flash lamps, white (200–1100 nm) and UV (200–400 nm) light	1 cycle (2 treatments)	Sublethal/ lethal	None	Yes
[48]	*L. monocytogenes* *L. innocua* *E. coli*	Xenon flash lamp (SteriPulse system), 200–1100 nm	10 cycles	No data	Single colony survivors replated on fresh solid medium and used for preparation of inoculum for next cycle	No
[44]	*L. monocytogenes*	SteriPulse-XL RS-3000C (xenon) pulsed light device	20 cycles	Lethal	TSA + YE plates with single colony survivors used for inoculation	Not stable tolerance (declined after deep-freeze storage)
**Viruses**						
[66]	Bacteriophage T7	UVP® transilluminator (bulbs, UVP 34-0042-01)	30 cycles	Lethal	A lysate of phage survivors added to a culture of cells and grown to culture lysis	Yes (resistance)

## 3. Biofilm Tolerance

Research on the possible phototreatment tolerance development were performed primarily using planktonic cultures. Nevertheless, one should remember, that biofilm growing bacteria substantially differ from their planktonic counterparts according to their susceptibility to antimicrobial agents [67]. Numerous studies reveled that eradication of bacterial biofilms required the administration of antimicrobials in concentrations 100–1000 times higher comparing to planktonic cultures [68,69]. Similarly, in the case of phototreatments, biofilm growing bacteria displayed lower susceptibility to the treatment, and using more rigorous conditions, i.e., increased PS concentration or light exposure, was required to reach the same antimicrobial effect [70]. The observed tolerance of biofilm growing microorganisms may result from the following issues: (i) extracellular polymeric substances (EPS) form the biofilm matrix acting as an antidrug-diffusion barrier [71]; (ii) biofilm growing cells display enormous genetic variation leading to adaptation to unfavorable environmental conditions [72] including oxidative stress [73]; (iii) altered genes expression. A comparative transcriptome analysis performed by Shemesh et al. [74] and Lo et al. [75], revealed that approx. 12% or 18% of genes, respectively, display a significantly different expression pattern when comparing planktonic vs biofilm growth. Changed expression was reported mainly for genes related to energy production, DNA replication and protein transport [74,75]. Moreover, within the microbial biofilms, persister-like cells are present, what may further complicate the tolerance development [76].

**Figure 4:**
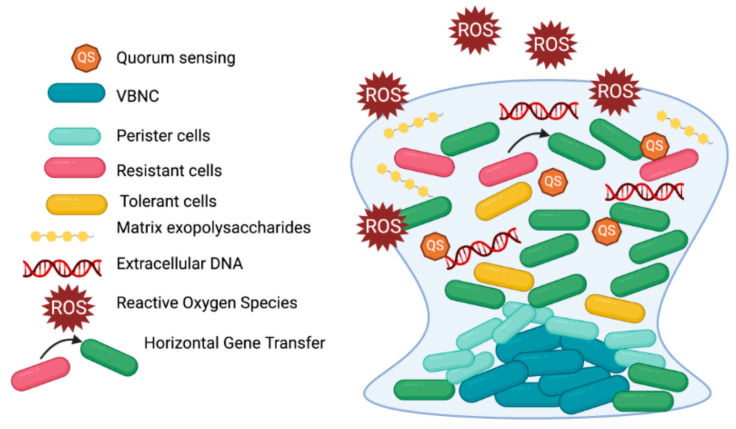
Schematic overview of a mature biofilm structure. Biofilm is characterized by heterogenous environment and the presence of a variety of subpopulations. A biofilm structure is composed of metabolically active (both resistant and tolerant) and non-active cells (viable but not culturable cells, VBNC, and persisters) as well as polymer matrix consisting of polysaccharide, extracellular DNA and proteins. Biofilm growth is associated with an escalated level of mutations and horizontal gene transfer (HGT) which is promoted in due to the packed and dense structure. Bacteria in biofilms communicate by QS, which activates genes participating in virulence factors production (modified from Hall and Mah).

Free-swimming cells are exposed to relatively homogeneous environment, while in case of biofilm growing cells, local chemical conditions (e.g., concentration of nutrients or metabolic waste) vary from each other in time and space [77]. It results in enormous heterogenicity and presence of variety of microbial subpopulations within the biofilm. Consequently, a susceptibility testing in biofilm, when applied for biofilm as a whole and not for separated subpopulations, is considered hardly possible [78] (Figure 4). The term ‘tolerance’ in case of biofilm could be defined as ability to survive in the presence of bactericidal agent and measured by method described previously (Figure 2) [5]. The term ‘resistance’ may be defined as capacity of cells to grow constantly in the presence of bacteriostatic or bactericidal agent in concentrations higher than MIC [79]. Although biofilms are responsible for over 65% of nosocomial infections, resistance is typically measured in planktonic cultures and the only country that approved biofilm susceptibility testing for clinical use is Canada [80]. 

It is generally accepted that the biofilm-specific antimicrobial tolerance and/or resistance is a multifactorial process and the spectrum of molecular adaptation depends on the microbial species and strain, growth conditions, stage of biofilm maturation and type of antimicrobial agent [81]. Study by Hall and Mah suggested that carefully performed experiments could make it possible to distinguish the mechanisms being involved in biofilm tolerance or resistance, however, it is not a trivial issue as numerous contradictory data are being published in this matter [78]. In fact, biofilm is a complex structure that involve a variety of tolerance and resistance mechanisms; thus, a new term “recalcitrance” was coined to depict the decreased sensitivity of biofilms to antimicrobials [82]. 

It is well known that persister cells are able to evade antimicrobial treatment in planktonic cultures [83]. Nevertheless, persisters are also considered an important factor in antibiotic tolerance of biofilms. The sub-MIC antibiotics and other external environmental stress factors results in biofilm enrichment for tolerant persister cells [76]. Moreover, study by Stewart et al. indicate that the existence of numerous phenotypes displaying various antibiotic tolerance may affect biofilm matrix composition, and finally, lead to different antimicrobial penetration within biofilms [84]. The antibiotic pressure in combination with the oxygen and nutrient shortage, can force the biofilm cells to enter a persister state enabling them to survive the lethal antimicrobial treatment [76]. The research performed by Spoering and Lewis demonstrated that the majority of P. aeruginosa biofilm-stage cells are as susceptible to antibiotics as planktonic counterparts and the increased tolerance could results from the higher amounts of persister cells recovered [85].

Another important issue is that biofilm growth promotes escalated mutability rate in cells. In studies by Ryder et al., the mutability of biofilm cultures increased 60-fold for *S. aureus* in comparison to planktonic counterparts [86]. Consequently, the biofilm stage can contribute to increased level of hypermutable strains [86,87]. The frequency of rifampicin- and mupirocin-resistant *S. aureus* mutants was higher in biofilm than in non-adherent cells [86]. Correspondingly, the mutation rate of ciprofloxacin resistant mutants was approx. 2-log higher in *P. aeruginosa* biofilm than in planktonic cultures [87]. In addition, Boles and Singh indicated that cells upbuilding biofilms are inherently prone to spontaneous mutations as a result of increased oxidative stress contributing to DNA damage [88].

Reactive oxygen species can be beneficial to the longevity through the mechanism of adaptation which is called “hormesis”. During this stage cells are exposed to low dose of stress and the induction of mechanism of protection is activated, thus, the cross adaptation to the other stresses can be developed. Concerning *P. aeruginosa* biofilms, their genetic variability results from the oxidative stress-induced DNA double strand brakes and also from the involvement of RecA, which activity leads to alterations in bacterial genome [73]. Experiments performed by Jakubowski and Walkowiak proved that the response to oxidative stress in planktonic cultures varies significantly from this occurring in biofilms [89].

Another important issue that could contribute to elevated biofilm tolerance to antimicrobial treatments is horizontal gene transfer (HGT). The phenomenon of HGT is promoted in biofilm due to the packed and dense structure and can be accomplished during conjugation, transformation or transduction [79]. Few studies suggested that plasmid transfer (via conjugation) could be more efficient in biofilm comparing to free-swimming counterparts. Studies performed by Savage et al. demonstrated that in case of *S. aureus* the conjugal transfer frequency of resistance plasmids was 16,000-fold increased than in planktonic cells [90]. Moreover, in experiments conducted by Cook and Dunny, the increased number of plasmid copies was observed, what resulted in elevated transcription level of plasmid-borne antibiotic resistance genes [91]. In addition, research performed by Strugeon et al. showed that the frequency of antibiotic resistance gene cassette was approx. 100-fold higher than in planktonic cultures [92].

Comprehension of the mechanism underlying biofilm-specific antimicrobial tolerance and resistance could significantly support the development of successful treatments that would overcome these mechanisms.

Despite increased biofilm tolerance to phototreatment, light-based strategies exert high antibacterial efficacy towards biofilm growing microorganisms. Cieplik et al. described the aPDI effectiveness towards *E. faecalis* biofilm formed for 72 h in 96-well polystyrene culture plates reaching significant reduction in cell viability (≥ 5 log_10_ CFU) [93]. In addition, a study by Biel et al. demonstrated that MRSA biofilms grown in an anatomically accurate maxillary sinus model (mimicking in vivo conditions of chronic rhinosinusitis, CRS) were susceptible to MB aPDI treatment displaying cell survival reduction by approx. 4 log_10_ CFU [94]. Similar cell viability reduction (approx. 4 log_10_ CFU) was observed for biofilms of ESBL-producing *Klebsiella pneumoniae* upon aPDI treatment [95]. In 2016 Halstead et al. using aBL reported *Acinetobacter baumannii* inactivation by approx. 1.5 log_10_ units when grown in biofilm culture [96] and this efficacy was further enhanced by Wang et al. who employed aBL against mature (24 and 72 h old) *A. baumannii* biofilm reaching the cell viability reduction by approx. 3–4 log_10_ [97]. Finally, significant antibiofilm activity was presented by Orlandi et al. who demonstrated 4 log_10_ reduction in viable cells growing in biofilm when exposed to aPDI [98].

## 4. Discussion

The phenomenon of resistance to antimicrobials has been known for years as a cause of treatment failure. As a result of an adaptive evolution, microorganisms under strong environmental stress change phenotypically and genetically to enable individuals to survive unfavorable conditions. The genetic basis of resistance to a particular antibiotic, clinically manifested as increased MIC values, is usually very well described and easily measured. When it comes to “resistance” to aPDI or aBL, the situation seems to be much more complicated due to their multitarget action, which mostly depends on the photosensitizing molecule used and its properties or localization. The low “resistance” rate that can be acquired by the action of a PS activated by light is a derivative of its ROS-driven, multitarget mode of action. However, there seems to be uncertainty about the development of “resistance” to aPDI and aBL treatment. Only some studies were able to demonstrate increased survival under lethal aPDI or aBL conditions. The discrepancies come from variations in methodology, various light–PS combinations, and that multiple microorganisms are under investigation. The available literature on aPDI and aBL shows that the development of resistance to this form of antimicrobial treatment is incomparably lower than for traditional antibiotics. Part of this is related to the multitarget action.

Nevertheless, it is not impossible to select a population of bacteria with reduced susceptibility to aPDI or aBL. The lack of success in this matter may, to some extent, be due to the analysis system being non-unified. While analyzing the action of an antibiotic on bacteria, they are under constant antibiotic pressure. In the case of a PS, although it may be present in the environment, enabling its constant exposure, its presence is still neutral. It acquires toxic properties only after exposure to light, which takes place only for a relatively short time. This might result in only brief selection pressure and potential consolidation of the aPDI- or aBL-treated population with a statistically low number/percentage of bacteria. Isolating this percentage of bacteria requires more extended tracking/observation or “enrichment” methods.

The current review was to provide the critical discussion over existing published studies concerning the possible development of tolerance/resistance to various light-based approaches (in particular aPDI mediated with various PSs classes and aBL, but also UV, CAP and PL treatments). In our opinion, the observed variations in methodological aspects are the reason explaining why, despite numerous existing studies, the phenomenon of tolerance was only demonstrated for a very limited number of researches. In our previous study [31], taking into account the protocols commonly used for tolerance/resistance testing against antibiotics [39], botanical antimicrobials [37] and microbiocides [38,41], we have designed and applied the protocol which enables successful testing of tolerance/resistance to various light-based approaches. We have identified the key issues that should be addressed to ensure an adequate tolerance testing. First of all, the sub-culturing for next consecutive cycles should originate from the treated bacterial suspension. In contrast, originating the overnight culture from a single surviving colony is burdened with the great risk of experimental failure due to the fact that the majority of the surviving bacterial cells, that may carry genetic alterations, are not included in the next cycle. Single surviving colonies were used as an inoculation source for the next cycle in numerous studies [19,24,27,29,30,32]. In none of them the phenomenon of a stable tolerance was observed. Only in case of research by Leanse at al., the increased unstable aBL tolerance in 9th, 16th and 17th cycle of aBL treatment could be demonstrated and suggested to result from persistence [29]. Treated suspensions were used for re-inoculation in six studies [20,26,28,31,33,35] and in case of three of them the stable tolerance was reported indicating that the population size used for re-inoculation may exert significant effect on adaptation process, due to the fact that the smaller populations typically maintain smaller amounts of genetic variations [65]. The next key issue that definitely should be addressed for tolerance testing is the treatment dose expressed as the reduction in viable counts. One should remember, that the application of too rigorous light-based treatment conditions could lead into irreversible changes in bacterial cells contributing to limited recover ability, and finally, not possible detection of tolerant phenotypes. Reviewed studies describe the use of both, lethal [19,20,21,25,29,32,36] as well as sublethal [18,24,26,27,28,30,31,33] treatment conditions. According to our observations, an exposure of examined strains to phototreatments should result in viability reduction by approx. 1–2 log_10_ units in viable counts to leave sufficient survivors for possible tolerance development; thus, the applied treatment conditions should be rather close to MDK_90–99_ parameter [9,37].

The next important issue that could affect the tolerance detection is the number of consecutive cycles. We have previously shown that in case of *S. aureus*, the tolerance development could be detected starting from the 4th–5th cycle [31]. However, in case of *S. agalactiae*, the aPDI tolerance could be demonstrated after completion of 10 treatment cycles [33], what indicates the need for experiment to be performed up to 15–20 cycles of sequential sub-culturing and treatment [37,40]. Moreover, within the designed protocol, we encourage to employ the stability testing enabling determination whether the observed adaptation results from the genetic changes and display stable phenotype.

All the studies that demonstrated the tolerance development meet the following requirements:

- the subculture originated from the treated suspension (not from a single surviving colony),
- the treatment condition resulted in the reduction in viable counts that left sufficient survivors for tolerance development (approx. 1–2 log_10_ units reduction),
- the experiment was conducted up to 7–15 cycles,
- phenotypic stability testing was performed.

Why methodology matters could be easily demonstrated by a pair of experiments conducted by Pedigo et al. and Snell et al., respectively. The experiments are linked by several factors: the same strain was included in the research (*S. aureus* ATCC 25923), the same PS (MB) and light source emitting overlapping wavelengths (670 nm and 575–700 nm, respectively). The main difference in methodology is that Pedigo et al. selected single colonies from solid medium formed upon aPDI cycle [18], while Snell et al. used a treated suspension for inoculation of overnight culture. Although the first group used sublethal doses of MB-aPDI treatment, as single colonies from a Petri dish were used for re-inoculation, thus there is a risk that treated cells with potential genetic alterations were not included in the next cycle.

Cold atmospheric plasma studies [50,54,58] did not reveal any bacterial adaptation development after consecutive treatments. In our opinion it may result from not appropriate inoculation source, i.e., single surviving colonies from solid medium, rather lethal than sublethal treatments used, or relatively small number of consecutive cycles, i.e., 4–6 cycles, described in majority of reviewed studies.

According to Ultra Violet light, it is commonly known that it exerts strong mutagenic and a lethal activity for a wide range of living organisms. It results in widespread DNA, proteins and cell membranes damage. UV is also an important selection and evolutionary factor as the early microbial life was exposed to potentially lethal doses of UV. In response to UV radiation bacteria have developed a number of repair pathways and mechanisms [99,100,101]. In the course of deep insight into UV-related studies reviewed within the current paper, we have identified that the requirement for suspension re-inoculation may not always be mandatory to obtain a UV tolerant/resistant bacterial subpopulation. In research conducted by Goldman and Travisano, bacteria were irradiated on solid media, incubated for 24h, resuspended and replated. The study revealed that *E. coli* K12 strain after cyclic sublethal UV treatment demonstrated essential adaptation to UV radiation. Moreover, it has been reported that UV-tolerant cells were characterized by increased cell size [65]. Increased cell size that is commonly observed in response to UV exposure [102] is considered to be a possible mechanism leading to reduced deleterious effect of UV radiation [103,104]. Davis and Sinskey (1973) observed that *Salmonella* Typhimurium subjected to repeated UV exposure resulted in increased cell size which contained approx. twice as high levels of RNA and proteins without altering the amount of DNA. In contrast, Alcantara-Diaz et al., performed studies using treated suspension for re-inoculation and also detected significant UV resistance development [64]. In case of study by Heinrich et al. the lethal treatment was employed that resulted in time dependent tolerance development [44]. Detected tolerance decreased after the deep-freeze storage; thus, we assume that the observed adaptation should be rather considered persistence and stability testing, i.e., passaging without selective pressure should be performed to distinguish between these two phenomena [44]. Finally, no adaptation was observed as a result of experiments conducted by Gomez-Lopez et al. (2005) and Uesugi et al. (2013) who also used single surviving colonies for the next cycles [46,48].

Abovementioned issues indicate that addressing the key elements of proposed protocol for tolerance/resistance testing in light-based therapies should be considered crucial, nevertheless, in case of UV-related studies a development of adaptation could also be reported without strictly following the proposed protocol.

The phenomenon of tolerance and its variability, known as persistence, further complicates the matter. Distinguishing between the three phenomena of resistance, tolerance, and persistence is not a trivial matter. Only recently has this begun to be appreciated in the context of clinical conditions regarding methods of assessing the clinically significant state of the pathogenic bacterial response to antimicrobial agents. Regarding light-based treatments, this approach has never been systematically verified and proposed as a method to describe increased bacterial survival after successive phototreatment cycles. From the available data in the literature, it appears that there is a phenomenon of reduced photokilling efficiency of bacterial cells, which is interpreted as tolerance to aPDI or aBL. We suggest that, similarly to the treatment of bacterial cells with antibiotics, with approaches based on the action of reactive oxygen (aPDI, aBL, CAP) or nitrogen (CAP) species, the nomenclature and the method of analyzing the tolerance phenomenon should be standardized. The MDK value, applied for the analysis of antibiotic tolerance, seems to be a suitable and straightforward measure for the tolerance phenomenon in photobiology studies. This measure may help in comparing the results obtained by different research groups.

With aPDI or aBL, we are not talking about resistance because, unlike the key–lock rule of antibiotic action, the mechanism by which light-activated compounds work is nonspecific. Thus, the primary mechanism underlying the phenomenon of increased survival upon treatment with aPDI or aBL described in the literature might, in fact, be tolerance. However, although increased survival upon sublethal treatment has been commonly observed, the mechanism explaining this observation remains obscure. As for now, no tolerant mutation has been identified upon light-based treatment, although there were reasonable indications that an aPDI- or aBL-induced tolerant mutant could be isolated as a result of SOS system activation under such treatment, which would further lead to elevated expression of error-prone polymerase mRNA [105].

From the in vitro and in vivo experiments on aPDI efficacy, the general problem is that if the bacteria survived treatment, after 12–24 h, regrowth of the whole population would be observed as a result of incomplete killing. This regrowth indicates that a subpopulation of cells could survive the treatment even though the conditions were harsh [106,107]. The observed survival strategy may be attributed to a persistence phenomenon that has not been studied in terms of aPDI or aBL but has been known to occur in response to antibiotics [108]. Among a clonal population of bacteria, typically less than 1% can survive high antibiotic concentration [17]. This small population can be described as time-dependent persisters as compared to dose-dependent persisters [5]. If such heterogeneity of a bacterial population were observed under aPDI or aBL, it would be of interest to determine whether a tolerant phenotype characterized those persisters. Another critical question has to do with identifying the molecular pathways that lead to a tolerant phenotype induced by a given phototreatment. Systematic classification of which phototreatments would be more characterized by dose-dependent or time-dependent persistence requires systematic analysis of bacterial responses to particular treatment types.

Taking into account the dependencies described in the latest reports on the sensitization of antibiotic-resistant microorganisms by treatment with aPDI or aBL, and placing these observations against the background of tolerance, persistence, or resistance observed in microorganisms, it seems to be a fascinating and necessary issue that has not been thoroughly explored. An interesting research path appears to be the characterization of individual types of light-based treatments, or other treatments based on an ROS-dependent mechanism, in the context of generating tolerance or induction in persisters. Do such processes occur in the phototreatments studied by us and others? Do they depend on the photosensitizing compound, the light dose, other light parameters, or a combination of these? Another critical issue is whether the observed phenomena of tolerance and persistence that are characterized in vitro by specific mathematical models and parameters, like MDK values, are relevant in vivo. For example, based on a study of *S. aureus*, the strains identified as tolerant to antibiotics (MDK value = 24 h) in vitro were most effectively killed by a longer treatment duration in vivo, rather than a higher antibiotic concentration [109]. Whether these phenomena apply to light-based treatment and ROS-dependent action of photosensitizing compounds should be studied.

## Data Availability

Data sharing not applicable.
